# Charting the progression of disability in parkinson disease: study protocol for a prospective longitudinal cohort study

**DOI:** 10.1186/1471-2377-10-110

**Published:** 2010-11-03

**Authors:** Leland E Dibble, James T Cavanaugh, Gammon M Earhart, Terry D Ellis, Matthew P Ford, Kenneth B Foreman

**Affiliations:** 1 Department of Physical Therapy, University of Utah, 520 Wakara Way, Salt Lake City, UT 84108, USA; 2 Department of Physical Therapy, University of New England, USA; 3 Program in Physical Therapy, Washington University in St. Louis-School of Medicine, USA; 4 Department of Physical Therapy and Athletic Training, Boston University, USA; 5 Department of Physical Therapy, School of Health Professions University of Alabama at Birmingham, USA

## Abstract

**Background:**

People with Parkinson disease (PD), even in the presence of symptomatic relief from medical, surgical, and rehabilitative interventions, face a persistent worsening of disability. This disability is characterized by diminished quality of life, reduced functional mobility, declining performance in activities of daily living and worsening neurological impairments. While evidence has emerged supporting the clinically meaningful benefits of short-term exercise programs on these underlying factors, assertions regarding the effects of sustained programs of exercise and physical activity on the trajectory of disablement in PD are made in the absence of direct evidence. Indeed, the natural decline in quality of life and functional mobility in people diagnosed with PD is poorly understood. Moreover, outcome measures commonly used in clinical exercise trials typically do not capture the full spectrum of disability as defined by the World Health Organization (WHO).

**Methods/Design:**

The objective of this multicenter prospective study will be to examine the 2-year trajectory of disablement in a cohort of persons with PD. Two hundred sixty participants will be recruited to produce an expected final sample size of 150 individuals. Participants will be included if they are greater than 40 years of age, have a neurologist confirmed diagnosis of idiopathic PD, and are at Hoehn and Yahr stages 1 through 4. Data will be collected every 6 months during the study period. Primary outcome measures reflecting a broad spectrum of disablement will include, but will not be limited to, MDS-UPDRS, Timed Up and Go, Berg Balance Test, Nine Hole Peg Test, PDQ-39, and directly monitored ambulatory activity. Self-reported exercise and physical activity data also will be recorded. Statistical analyses will be used to characterize the trajectory of disablement and examine the influence of its underlying contributing factors.

**Discussion:**

Tertiary prevention is an important component of contemporary healthcare for individuals living with degenerative disease. For individuals with PD, there is growing recognition that exercise and/or physical activity efforts to slow the rate of functional mobility decline, in particular, may be critical for optimizing quality of life. By describing the natural trajectory of disablement, exercise habits, and physical activity in a cohort of persons with PD, this investigation will establish an important foundation for future intervention research. Specifically, through the evaluation of the influence of sustained exercise and physical activity on disablement, the study will serve as a preliminary step toward developing a randomized controlled trial of long-term exercise in persons with PD.

## Background

Parkinson disease (PD) is a chronic, progressive neurodegenerative disease affecting more than 4 million people world-wide[1]. With adequate access to healthcare services, persons with PD can live 20-30 years following initial diagnosis[2]. Nonetheless, persons with PD face a persistent deterioration in functional mobility and activities of daily living often resulting in a loss of independence and a decline in quality of life.

Over the last decade, a growing body of evidence has emerged revealing significant and clinically meaningful benefits of exercise for addressing PD-related problems. For example, a critical review of the literature identified 23 randomized controlled trials demonstrating that patients who participated in exercise programs had better quality of life, walking ability, balance, strength, flexibility and cardiovascular fitness compared to those who did not exercise[3]. Exercise studies of both rodent and primate models of PD have demonstrated increased survival of nigrostriatal dopaminergic neurons, suggesting a potential protective effect of exercise as well[4,5]. Furthermore, a prospective epidemiological study revealed a significant decreased risk of developing PD in people who participated in moderate to vigorous exercise[6,7].

Although promising, studies of exercise in PD have been limited in scope. Most have examined the effects of short-term exercise programs, typically implemented over 4 to 12 weeks. Furthermore, in studies with prolonged follow-up, exercise benefits typically attenuated over weeks to months following the intervention period[8,9]. Thus, the benefit of longer, more sustained patterns of regular exercise on PD-related problems remains poorly understood. In particular, the impact of sustained exercise and/or a physically active lifestyle on the rate at which persons with PD become disabled (i.e., the "trajectory of disablement") remain unknown.

Currently the Unified Parkinson Disease Rating Scale (UPDRS) and its most recent version (the MDS-UPDRS) are considered the gold standards for examining disease severity and progression[10-12]. The UPDRS focuses primarily on measuring impairments associated with PD, with fewer items addressing specific functional limitations or perceptions of quality of life. The subsections of the UPDRS are organized according to motor and non-motor aspects of the disease, significantly limiting the assessment of disablement in PD. As a result, the burden of PD is commonly understood more in terms of disease progression (i.e., the predictable evolution of signs, symptoms, and impairments) rather than in terms of the potentially diverse paths through which persons with PD become disabled.

In contrast to the UPDRS, the International Classification of Functioning, Disability, and Health (ICF) was developed as a framework for understanding disability at multiple levels[13]. Accordingly, the effect of health conditions (e.g., PD) is considered across 3 domains of human function: body structure and function, activity, and participation. "Disability" is used to denote a decrement at each level (i.e., a body structure or function impairment, an activity limitation, and a participation restriction)[13]. Underscoring the value of this approach, the World Health Organization (WHO) endorsed the ICF in 2001[14].

"Body structure," in ICF terms, is defined as an anatomical part of the body, such as organs, limbs and their components, while "body function" is defined as the physiological function of body systems. Applied to PD, motor signs such as bradykinesia, tremor and rigidity represent impairments in body structure and function. "Activity" is defined as the execution of a task or action by an individual and activity limitations as the difficulties an individual may have in executing such tasks. Activity limitations common in PD are those affecting gait, balance, getting dressed, bathing, and other activities of daily living. Lastly, "participation" is defined as the involvement in a life situation and participation restrictions as problems an individual may experience in involvement in life situations. Participation restrictions in PD may include involvement in leisure, work or social aspects of life in both the household and community settings[14].

To address the aforementioned limitations in the exercise and disability literature, we plan to conduct a 2-year, multicenter prospective longitudinal study of a cohort of persons with PD. The objectives of the study will be: (1) to characterize the natural trajectory of disablement using a spectrum of measures organized according to the ICF framework, and (2) to identify potential factors, including but not limited to those related to exercise and physical activity, that contribute to the development of impairments of body structure/function, activity limitations, or participation restrictions. The study is anticipated to lay an important foundation for future studies, especially those designed to understand the impact of sustained exercise and/or a physically active lifestyle on disablement in PD.

## Methods/Design

### Research Design

The proposed project is a prospective, longitudinal cohort study in which patients with PD will be assessed every 6 months over a 2-year period using a specific battery of outcome measures designed to reflect the full spectrum of disability.

### Participants and Recruitment

Two hundred sixty participants will be recruited through the Neurology Department at the University of Utah, the Parkinson's Disease and Movement Disorder Center at Boston University Medical Center (BU PDMC), the Department of Neurology at the University of Alabama at Birmingham (UAB), and the Movement Disorders Center at Washington University in St Louis School of Medicine. Prior to data collection, ethics approval will be achieved through the Institutional Review Board (IRB) at each participating site. Before participation, all participants will sign IRB approved informed consent forms. Gathering data at four sites will promote rapid enrollment and increase the likelihood of enrolling participants who typically are under-represented in PD studies (e.g., Ethnic minority participants, females, individuals who are at Hoehn and Yahr Stage 4). Included participants will be community dwelling persons ≥ 40 years of age who have neurologist-diagnosed idiopathic PD (using UK Brain Bank Criteria [15]), are at Hoehn and Yahr stages I-IV (mild to moderate disease severity), and score ≥ 24/30 on the Mini-mental State Examination. Excluded participants will be individuals with a diagnosis of atypical Parkinsonism, who are at Hoehn and Yahr stage 5, or who have had previous surgical management of PD (e.g. pallidotomy, deep brain stimulation).

### Power and Sample Size Estimates

A 25% annual attrition rate is anticipated over the 2-year study period, resulting in a final estimated sample size of 150 participants. The final sample will be sufficient to provide precise disability estimates and to allow for the inclusion of as many as 12 predictor variables in multiple regression analyses that evaluate the underlying contributors to various trajectories of disablement (α = 0.05; N ≥ 50 + 8 (X), where X = the number of predictor variables.)[16]

### Procedures

**Figure 1:**
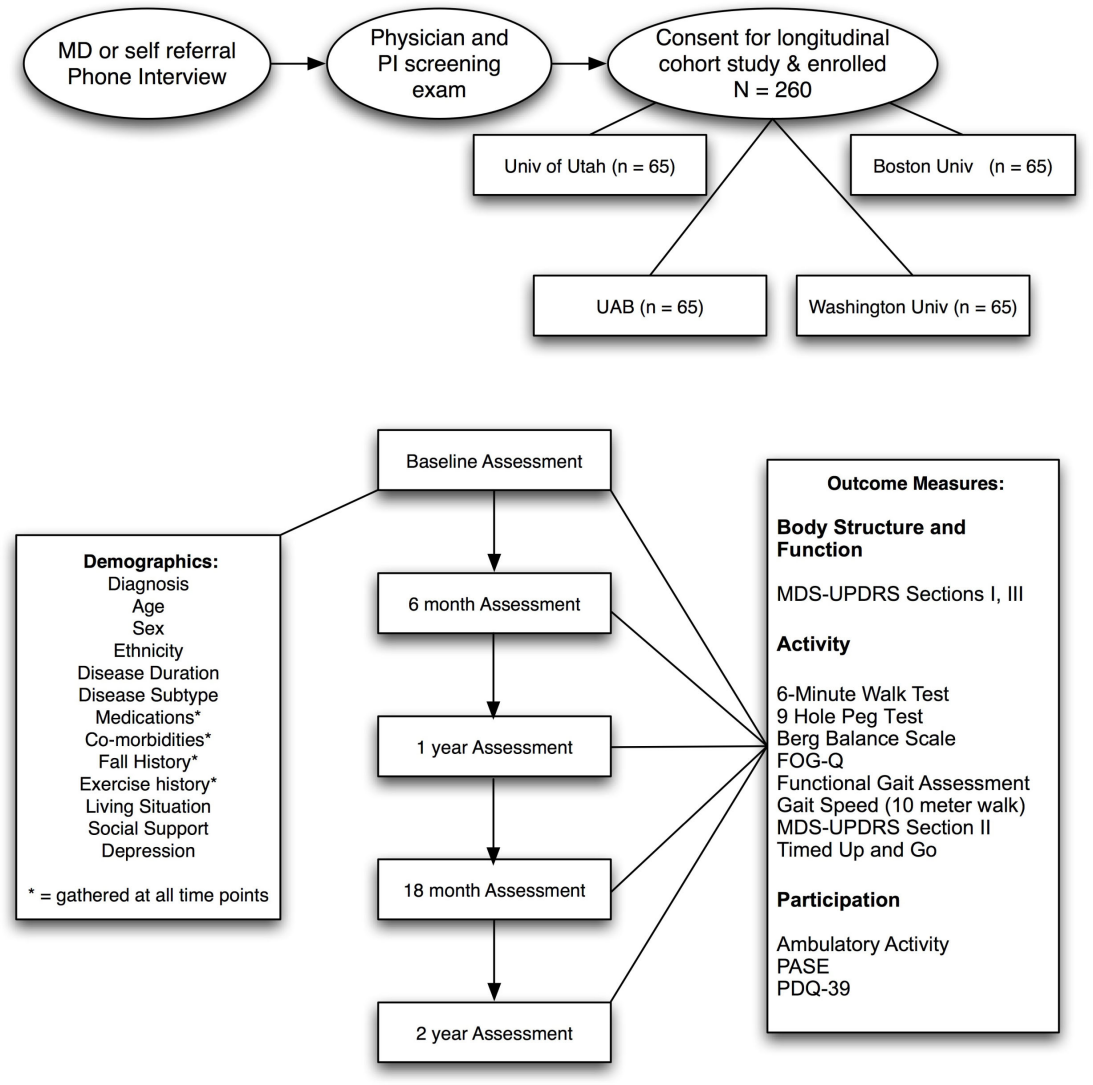
**Flow sheet of Study Procedures**. Abbreviations: UAB = University of Alabama at Birmingham, MDS-UPDRS = Movement Disorders Society-Unified Parkinson Disease Rating Scale, FOG-Q = Freezing of Gait Questionnaire, PDQ-39 = Parkinson Disease Questionnaire - 39, PASE = Physical Activity Scale for the Elderly.

At baseline, demographic information and disease history will be collected via interview. At each measurement interval (i.e., baseline, 6 months, 1 year, 1.5 years and 2 years), (1) co-morbidity data will be collected using a modified version of the Comorbidity Questionnaire, a reliable and valid instrument that offers practical advantages over medical record based assessments [17]; (2) depression will be assessed using the Geriatric Depression Scale [18,19]; and (3) medication data (i.e., drug name, dose, frequency, levo-dopa equivalence) will be collected using a customized form. In addition, a suite of standardized instruments will be employed at each measurement interval to capture participant physical function, mobility, physical activity, and quality of life. The instruments have been selected for their strong psychometric properties and collective representation of ICF domains (Table 1 and Figure 1)

**Table 1:** Battery of Outcome Measures

ICF Disablement Construct	Outcome Variable
Body Structure and Function	
	MDS-UPDRS Sections I, III

Activity	
	6 Minute Walk Test
	9 Hole Peg Test
	Berg Balance Scale
	Freezing of Gait Questionnaire
	Functional Gait Assessment
	Gait Speed (10 meter walk)
	MDS-UPDRS Section II
	Timed Up and Go

Participation	
	Ambulatory Activity
	PASE
	PDQ-39

MDS-UPDRS = Movement Disorders Society-Unified Parkinson Disease Rating Scale; PASE = Physical Activity Scale for the Elderly; PDQ-39 = Parkinson Disease Questionnaire - 39

### Measure of Body Structure and Function

Sections I and III of the MDS-UPDRS will be utilized to assess neurological signs[10,12]. Section I, which is entitled "Non-motor aspects of experiences of daily living", consists of 13 items that will be administered via clinician interview and a participant/caregiver questionnaire. Section III consists of 18-items pertaining to motor aspects of the disease, the data for which will be obtained by examining the participant at the time of each visit. Each item is rated on a 5-point (0-4) ordinal scale, with higher scores indicating more severe impairment.

### Measures of Activity

(1) Section II of the MDS-UPDRS consists of 13 items related to activities of daily living (ADL). Data will be obtained via interview. Each item is rated on a 5-point (0-4) ordinal scale with higher scores indicating more severe activity limitation. The UPDRS has been administered in several large clinical trials in PD and is reliable, valid and responsive to change[10,12].

(2) The Berg Balance Scale (BBS) is a 14-item test battery that quantitatively assesses balance and risk for falls through direct observation of performance[20]. The BBS requires approximately 15 minutes to complete and measures the participant's ability to maintain balance either statically or dynamically over a specified period of time[21]. The items are scored on a 5-point (0-4) ordinal scale. The total score, which will be used as the dependent variable, ranges from 0 to 56 with higher scores indicating better balance. Validity and high test-retest reliability of the BBS total score have been demonstrated across a variety of populations including patients with PD[22,23].

(3) The Functional Reach Test (FR) measures the maximum distance a participant can reach in the forward direction with a fixed base of support[24]. Each participant will be asked to make a fist, raise the dominant arm parallel to the floor and reach as far forward as possible without taking a step. Using a yardstick mounted on the wall at the shoulder height and the third metacarpal as the reference point, the distance between the starting and ending position will be recorded. Two practice trials and 3 test trials will be conducted, with the mean distance reached during the 3 test trials used as the dependent variable. Validity and high test-retest reliability of the FR have been established in healthy elders and in people with PD [23,24].

(4) The Functional Gait Assessment (FGA) is a 10-item standardized test for assessing postural stability during various walking tasks. Items include walking with head turns, walking with eyes closed, walking while altering gait speed, walking in a backward direction, walking with a narrowed base of support, negotiating obstacles, stopping, turning and stair climbing. Items are scored using a 4-point ordinal scale (0-3). Total score, which will be used as the dependent variable, ranges from 0 to 30, with higher scores indicating better performance. Reliability, internal consistency and validity of the FGA total score have been established in healthy adults and in patients with neurological disorders[25,26].

(5) The six-minute walk (6MW) test, a measure of the distance a participant walks in 6 minutes, will be used to assess overall locomotor ability. The 6MW distance is related to functional movement tasks and is an independent predictor of prognosis in older patients with co-morbid conditions. The 6MW's test-retest reliability is high, ranging from .94 - .96, in older populations with various co-morbid conditions[23,27].

(6) Self-selected and maximal pace gait speed will be measured during a 10 meter walk. Three trials at each pace will be recorded, with the average speed at each pace used as separate dependent variables. Gait speed provides a standardized measure of gait function that has been found to be reliable and is sensitive to change over a broad range of physical function in elderly individuals and persons with neurologic pathology[23,27,28].

(7) The Freezing of Gait Questionnaire (FOG-Q) is a valid and reliable 6-item tool used to assess the severity of freezing of gait in patients with PD[29,30]. Each item is rated on a 5-point ordinal scale, from 0 (absence of symptom) to 4 (most severe symptom). The total score, which will be used as the dependent variable, reflects the sum of the 6 items and ranges from 0-24. The FOG-Q will be self-administered by all participants.

(8) The "Timed Up and Go" Test (TUG) measures the time it takes for a participant to stand from a seated position in an armchair, walk forward 3 m at a comfortable pace, turn around, walk back to the chair, and sit down. Each participant will perform a practice trial followed by 2 test trials. The mean of the 2 test trials will be the dependent variable. In older adults at risk for falls, the TUG has been found to possess excellent intra and inter-tester reliability (.94-.96) and predictive validity in that increased times on the TUG relate to increased fall-risk[23,31,32].

(9) The Nine Hole Peg Test (9-HPT) is a brief, standardized, quantitative test of upper extremity function that asks the participant to place and remove nine pegs one at a time, as quickly as possible, from nine holes in a peg board. Scoring is determined by the total time to complete the task. Two trials with the dominant hand will be immediately followed by two trials with the non-dominant hand. The mean of the two trials for each hand will be the dependent variable. The 9-HPT has high inter-rater reliability and good test-retest reliability. There is evidence for concurrent and convergent validity as well as sensitivity to detect minor impairments of hand function[33,34].

### Measures of Participation

(1) The Parkinson's Disease Questionnaire-39 (PDQ-39) is a health status instrument that contains 39-self-report items and was specifically developed for people with Parkinson's disease. The PDQ-39 measures the degree of healthy, competent, and satisfying participation in daily life activities. The reliability, validity, and sensitivity to change of the PDQ-39 have been established in community dwelling persons with PD. In addition to the composite summary score, we will utilize the 8 subscores (i.e., mobility, activities of daily living, emotions, stigma, social support, cognition, communication, and body discomfort) to reflect constructs that have been consistently found to contribute to perceived quality of life in persons with PD)[35-37].

(2) Ambulatory Activity: To capture "free-living" ambulatory activity, a sub-group of participants will wear a StepWatch 3 Activity Monitor (SAM, Orthocare Innovations, Seattle, WA,), 24 hours per day for 7 consecutive days, except when bathing, showering, or swimming. The SAM is approximately the size of a pager, weighs 38 g, and is attached using Velcro closures immediately proximal to the lateral malleolus of either leg. The SAM uses a combination of acceleration, position, and timing to detect steps taken by the leg on which it is worn. It is designed for long-term use during daily activities performed in an individual's customary environment over hours or days without maintenance by the user. Data are recorded as a temporal series of counts, with each data point representing the number of steps per one-minute interval. Data will be downloaded to a personal computer via an infrared docking station and post processed using either manufacturer software or custom analysis programs written in Matlab (Mathworks, Natick, MA). Step counts will be used to generate multiple indices of mean daily ambulatory activity (e.g., total number of steps, percent of day spent inactive, total number of bouts of activity, bout duration, and activity intensity). The SAM is particularly accurate for individuals with impaired gait, is unobtrusive and easy to use, and has demonstrated good test-retest reliability (ICC, r = 0.84) and accuracy (98%) in an older adult population[38-42].

(3) The Physical Activity Scale for the Elderly (PASE) measures the level of self-reported physical activity in individuals aged 65 years or older and is comprised of items regarding occupational, household, and leisure activities during the previous 7-day period[43,44]. Leisure activities include frequency and duration of walking outside the home, as well as participation in light, moderate and strenuous exercise. The total PASE score, which will be used as the dependent variable, is computed by multiplying the time spent in each activity (hours/week) or participation (yes/no) in an activity by empirically derived item weights and summing over all activities. The PASE has previously been validated in elderly populations[45-47].

### Statistical Analysis Plan

Confidence intervals, means, standard deviations and frequency distributions will be calculated for all measures. Corrections for multiple comparisons will be used to control for increased type I statistical error risk. Effect sizes and post hoc power will be calculated when appropriate. All analyses will be performed with SPSS 16.0 (SPSS Inc).

To address study objective 1, disablement trajectory will be characterized using interval and point estimators of each outcome measure. In addition, survival analyses will be utilized to examine the time to reach operationally defined mobility thresholds (i.e., limited community ambulatory gait speed, assistance with ADL's, time to recurrent falls). This approach will allow us to characterize the distribution of time-to-event data, to test for differences between subgroups (i.e., exercise history, disease severity, disease sub-type, age, number of cormorbidities), and to utilize regression models to analyze complex influences of covariates on time to event data[16].

To address study objective 2, multiple regression analyses will be conducted to identify those factors associated with progression of disability. Assessments for violation of assumptions will be made, including analyses of normality of the residuals and linearity of the continuous variables[16]. As needed, potentially more potent contributors will be used to define subgroups within the sample (e.g., exercisers vs. non-exercisers; physically active vs. sedentary), and analyses of variance will be used to evaluate between- and within-group differences in disablement change.

### Data management

The multicenter protocol will rely on a web-based system of data input into a central database. The system will feature multi-tiered security-protected access and will conform to HIPAA security policies. Stored data will be backed up daily. Authenticated investigators will have access to the dataset from any Internet access point.

Prior to data collection, study personnel at each site will review a standard operating procedures (SOP) protocol manual and will rate two standardized persons with PD, who will have been filmed performing the battery of physical performance tests. Study personnel and site specific ratings will be evaluated by the data management team to insure their accuracy and consistency.

At each measurement interval, data will be collected on paper forms and entered into the web-based data entry portal by a specific individual at each site. The data management team subsequently will confirm the accuracy of data entry for every third participant by comparing electronic data with the original hard copy data. In addition, the team will select files at random for similar review. Researchers at each site will be notified of any discrepancies or incomplete data.

## Discussion

Tertiary prevention is an important component of contemporary healthcare for individuals with degenerative disease. Yet only recently has attention been given to the evolution of disability among persons with PD[13,48-51]. Such studies, however, while valuable, have been limited by cross-sectional designs and limited scope of disablement. To our knowledge, our proposed study is novel for its longitudinal examination of the natural trajectory of disablement, exercise habits, and physical activity in a cohort of persons with PD. In this context, data collected in the study will provide a foundation for future exercise intervention research. Not only will the study characterize disablement using a broad spectrum of measures, it also has the potential to identify factors that might influence, either positively or negatively, the rates at which persons with PD experience decline in mobility and quality of life.

## Competing interests

The authors declare that they have no competing interests.

## Authors' contributions

JTC, LED, GME, TDE, and MPF originally conceived the idea for the study. JTC, LED, GME, TDE, MPF and KBF all participated in the design of the study and selection of outcome measures. JTC conceived the idea for the ambulatory activity data collection component of the study and will provide data analysis and interpretation of this data. KBF conceived and implemented the database structure and the on-line based standard operating procedures manual and tester training. At their individual sites all authors provided project management and assisted with subject recruitment and safety monitoring. LED oversaw monitoring of data integrity and verification as well as wrote the initial draft of the manuscript for submission to BMC Neurology. All authors assisted in editing the final submitted manuscript. All the authors have read and approved the manuscript.

## Pre-publication history

The pre-publication history for this paper can be accessed here:

http://www.biomedcentral.com/1471-2377/10/110/prepub
